# Protective Effects of the Postbiotic *Lactobacillus plantarum* MD35 on Bone Loss in an Ovariectomized Mice Model

**DOI:** 10.1007/s12602-023-10065-7

**Published:** 2023-04-01

**Authors:** Ju-Yeong Myeong, Hye-Yeon Jung, Hyo-Seok Chae, Hyang Hyun Cho, Don-Kyu Kim, You-Jee Jang, Jae-Il Park

**Affiliations:** 1 Animal Facility of Aging Science, Korea Basic Science Institute, Gwangju, 61751 Republic of Korea; 2 Department of Integrative Food, Bioscience and Biotechnology, Chonnam National University, Gwangju, 61186 Republic of Korea; 3 MEDINUTROL Co., Ltd., Yeonggwang, 57024 Republic of Korea; 4 Department of Biomedical Laboratory Science, Honam University, Gwangju, 62399 Republic of Korea

**Keywords:** *Lactobacillus plantarum*, Postbiotics, Osteoclast, Osteoporosis

## Abstract

Postmenopausal osteoporosis is caused by estrogen deficiency, which impairs bone homeostasis, resulting in increased osteoclastic resorption without a corresponding increase in osteoblastic activity. Postbiotics have several therapeutic properties, including anti-obesity, anti-diabetic, anti-inflammatory, and anti-osteoporotic effects. However, the beneficial effects of the postbiotic MD35 of *Lactobacillus plantarum* on bone have not been studied. In this study, we demonstrated that the postbiotic *L. plantarum* MD35, isolated from young radish water kimchi, influences osteoclast differentiation in mouse bone marrow-derived macrophage (BMM) culture. In addition, it was effective protecting against estrogen deficiency-induced bone loss in ovariectomized (OVX) mice, an animal model of postmenopausal osteoporosis. In BMM cells, postbiotic MD35 inhibited the receptor activator of nuclear factor-kappa B of NF-κB ligand (RANKL)-induced osteoclast differentiation by attenuating the phosphorylation of extracellular signal-related kinase, significantly suppressing the resorption activity and down-regulating the expression of RANKL-mediated osteoclast-related genes. In the animal model, the oral administration of postbiotic MD35 remarkably improved OVX-induced trabecular bone loss and alleviated the destruction of femoral plate growth. Therefore, postbiotic MD35 could be a potential therapeutic candidate for postmenopausal osteoporosis by suppressing osteoclastogenesis through the regulation of osteoclast-related molecular mechanisms.

## Introduction

Bone is a dynamic organ in which osteoblast-mediated formation and osteoclast-mediated resorption are strictly regulated both temporally and spatially [1, 2]. An imbalance between osteoblasts and osteoclastic activity causes pathophysiological bone diseases such as osteoporosis, which can lead to decreased bone density and bone weakness. This process leads to deterioration in bone mass and microarchitecture with increasing fragility, which predisposes the bone to fractures [3, 4]. Osteoporotic fractures lead to acute pain, increased morbidity and mortality in the elderly, and ultimately, lower quality of life and higher healthcare costs [5]. Osteoporosis is a major health problem that has significantly increased in recent years. More than 75 million people worldwide and one in three women over the age of 50 years suffer from osteoporosis, 80% of which are postmenopausal [6]. In women, the two major causes of bone loss are menopause-induced estrogen deficiency and aging. Senile osteoporosis is related to aging and is caused by deficiencies in dietary calcium and vitamin D, and increased activity of the parathyroid glands [7]. Postmenopausal osteoporosis in women is associated with decreased estrogen production, which enhances the number and function of osteoclasts [8].

Menopause-induced estrogen deficiency is the most important cause of osteoporosis, as it enhances the number of osteoclasts and osteoclast formation [9]. Osteoclasts derived from the undifferentiated cells of a monocyte-macrophage lineage are multinucleated giant cells stimulated with two cytokines, macrophage colony-stimulating factor (M-CSF) and NF-κB ligand (RANKL) [10, 11]. M-CSF is essential for the survival and proliferation of osteoclast precursors, and RANKL controls the function and survival of mature osteoclasts by interacting with its receptor RANK [12–14]. Following RANKL-to-RANK interaction, tumor necrosis factor (TNF) receptor-associated factor 6 (TRAF6) is recruited immediately leading to the activation of multiple downstream signaling events, including the synthesis of NF-κB and mitogen-activated protein kinases (MAPKs), in the early stage of osteoclast differentiation [13–16]. In addition, various transcription factors, such as NF-κB, c-Fos, and nuclear factor-activated T cells c1 (NFATc1), responsible for osteoclast differentiation are activated [17, 18]. In particular, NFATc1, a key regulator of osteoclast differentiation, regulates several osteoclast-targeting genes such as cathepsin K (Ctsk), tartrate-resistant acid phosphatase (Trap), calcitonin receptor (Ctr), osteoclast-associated receptor (Oscar), and matrix metalloproteinase-9 (MMP-9), through cooperation with c-Fos [19–21]. Therefore, blocking the intercellular signaling and inhibiting the activations of osteoclast-specific genes stimulated by RANKL is a major therapeutic target for treating menopause-induced osteoporosis.

Some nutrition supplements (such as calcium, vitamin D, and vitamin K), selective estrogen receptor modulators (such as calcitonin, parathyroid hormone, and other estrogen analogs), bisphosphonates, and hormone replacement therapy, are used to reduce the risk of bone loss and fractures after menopause [9, 10]. Bisphosphonates, such as alendronate and zoledronic acid are the most widely prescribed medications; however, long-term administration may increase the incidence of osteonecrosis of the jaw bone and fracture of the femur, as well as upper gastrointestinal discomfort, venous thromboembolism, and cancer [11, 22]. Therefore, there is an urgent need to identify alternative treatments that have fewer adverse effects.

Probiotics are live strains of microorganisms that confer health benefits when administered in adequate quantities. Various types of probiotics, especially *Lactobacillus spp*., have anti-osteoporotic activities [23–27]. A milk product fermented by *L. casei* 393 significantly attenuated the reduction in bone strength that occurs in response to ovariectomy [24]. Femoral trabecular numbers are increased by *L. paracasei* (NTU 101) and *L. plantarum* (NTU 102)-fermented soy skim milk in ovariectomized (OVX) mice [25]. Probiotic treatment with either *L. paracasei* or a mixture of *L. paracasei* and *L. plantarum* protects mice from OVX-induced cortical bone loss and increased bone resorption [26]. Three probiotic strains, *L. acidophilus*, *L. reuteri*, and *L. casei*, ameliorate bone loss in an OVX-induced rat model [27].

Probiotics are safe for disease therapy; however, the use of live bacteria could be associated with severe infections and increased mortality [28]. A growing number of probiotic effects are imparted by postbiotics, which are microbial metabolites of probiotics that are particularly useful in maintaining gut health and curing gut diseases [29, 30]. The microbial metabolites of probiotics contain either soluble or secreted factors. Metabolites, bacteriocins, and cell-free supernatants have the same mechanism of action and capacity as probiotics because secondary metabolites of probiotics are present, however, there are no living cells [29, 30]. Therefore, postbiotics have several pharmaceutical properties beneficial to critically ill patients, young children, and premature neonates [31, 32].

Unlike probiotics, the anti-osteoporotic effects of postbiotics of *L. plantarum* remain unknown. Therefore, in this study, we investigated the inhibitory effects of postbiotic MD35 on osteoclast differentiation and established the mechanism underlying the RANKL-induced osteoclasts in vitro. An OVX mouse model was used to examine the protective role of postbiotic MD35 against bone loss and its inhibitory action on osteoclast differentiation in vivo. To the best of our knowledge, this is the first study to examine the effects of postbiotics of *L. plantarum* on bone health and homeostasis.

## Materials and Methods

### Reagents and Antibodies

Recombinant macrophage colony-stimulating factor (M-CSF) was purchased from R&D Systems, Inc. (Minneapolis, MN, USA) and recombinant RANKL was purchased from Abcam (Cambridge, MA, USA). For western blotting analysis, the following antibodies were used: c-Jun N-terminal kinase (JNK) (R &D Systems Inc.), Matrix metalloproteinase 9 (Mmp-9) (Abcam), c-Fos, p-p42/44, p42/44, p-AKT, and AKT (Cell Signalling Technology, Massachusetts, MA, United States); Gapdh, p38, and IκBα, HRP anti-mouse, and HRP anti-rabbit (Santa Cruz Biotechnology, San Diego, CA, USA); p-p38 and p-JNK (New England Biolabs, Ipswich, MA, USA); NFATc1 (Novus Biologicals, Littleton, CO, USA).

### Preparation of *Lactobacillus plantarum* for In Vitro Experiments

The *Lactiplantibacillus plantarum* V135 (KCTC18796P) strain from Korean fermented kimchi was isolated by Medinutrol Co., Ltd. (Yeonggwang, Republic of Korea) and preserved in the Korean Collection for Type Cultures (KCTC, Daejeon, Republic of Korea). Postbiotic MD35 consists of powdered dead-cell bacteria and a cultivated medium with dextrin as an excipient. The bacteria were cultivated for 24 h at 37 °C in modified MRS (de Man, Rogosa and Sharpe) broth replaced with edible ingredients to reach the bacterial concentration of 2 × 10^9^ CFU/mL The cultures were powdered using Spray dryer (JI tech, Republic of Korea) following heat treatment at 104 °C twice.

### Mouse Bone Marrow-Derived Macrophage (BMM) Culture 

BMMs were isolated from 6 − 8 week-old C57BL/6 mice using α-minimum essential medium (α-MEM, Gibco BRL, Gaithersburg, MD, USA) supplemented with M-CSF (5 ng/mL), 1% penicillin/streptomycin (GIBCO, Chagrin Falls, Ohio, USA), and 10% fetal bovine serum (FBS, Thermo Fisher Scientific Inc. Waltham, MA, USA). BMM were seeded into 10-cm dishes and cultured with M-CSF (30 ng/ml) for 3 days in a humidified atmosphere with 5% CO_2_ at 37 °C. After removing the floating cells, adherent cells were used in the experiments.

### Cell Viability Assay

BMM cells (5 × 10^3^ cells) were seeded in 96-well plates and incubated with the indicated concentrations of the postbiotic MD35 in the presence of M-CSF (30 ng/ml) for 3 days. Cell viability was assessed by a Quick Cell Proliferation Assay Kit (BioVision, Mountain View, CA, USA), and the absorbance was determined at 570 nm using a microplate reader (SpectraFluor, TECAN, Sunrise, Austria). Cell viability in the 0 μg/mL of MD35 was considered as 100%, and the percentage values for three different dosages (5, 10, and 50 μg/mL of MD35) were calculated.

### Osteoclast Differentiation and TRAP Activity Assay

To test the effect of MD35 on osteoclastogenesis, BMM cells (1 × 10^4^ cells) were seeded in a 96-well plate and cultured with M-CSF (30 ng/mL) and RANKL (100 ng/mL) with or without the indicated concentrations of MD35 for 7 days. After TRAP staining using a TRACP and ALP double-staining kit (Takara, Shiga, Japan), TRAP-positive multinucleated cells containing more than three nuclei were counted as osteoclasts. Microscopic images were captured and the number of nuclei per cell was counted using ImageJ. Absorbance was measured at 405 nm.

### Quantitative Real-Time Polymerase Chain Reaction (qPCR) Analysis

Total RNA was isolated using a Total RNA mini kit (Favorgen Biotech, Ping-Tung, Taiwan) according to the manufacturer’s instructions. Two micrograms of total RNA were used for cDNA synthesis and qPCR was performed on a Rotor-Gene Q 5plex (QIAGEN; located at the Korea Basic Science Institute in Gwangju, Korea) using the QuantiTect SYBR Green PCR Kit (QIAGEN, Hildon, Germany). The following primers were used for qPCR: Nfatc1 5′- CTCGAAAGACAGCACTGGAGCAT-3′ (forward) and 5′-CGGCTGCCTTCCGTCTCATAG-3′ (reverse); Oscar 5′-CTGCTGGTAACGGATCAGCTCCCCAGA-3′ (forward) and 5′-CCAAGGAGCCAGAACCTTCGAAACT-3′ (reverse); Ctsk 5′-ACGGAGGCATTGACTCTGAAGATG-3′ and 5′-GGAACCACCAACGAGAGGAGAAAT-3′ (reverse); Trap 5′-CTGGAGTGCACGATGCCAGCGACA-3′ (forward) and 5′-TCCGTGCTCGGCGATGCACCAGA-3′ (reverse); c-Fos 5′-CCAGTCAAGAGCATCAGCAA-3′ (forward) and 5′-AAGTAGTCGCAGCCCCGAGTA-3′ (reverse); Ctr 5′-TGGTTGAGGTTGTGCCCA-3′ (forward) and 5′-CTCGTGGGTTTGCCTCATC-3′ (reverse); Mmp-9 5′-CGTCGTGATCCCCACTTACT-3′ (forward) and 5′-AACACACAGGGTTTGCCTTC-3′ (reverse). The mean Ct value of three determinations for each gene was divided by the linear Ct of the β-actin (Actb) gene to obtain the relative abundance of the transcript. Mean values were obtained from three or four separate experiments. Actb was used as an internal control for all measurements.

### Bone Resorption Assay

Bone resorption activity was measured using a bone resorption assay kit (Corning Inc., Corning, NY, USA). BMM cells were seeded in a Corning Osteo Assay Surface 96-well plate at a density of 5 × 10^3^ cells/well and incubated in α-MEM with 10% FBS, M-CSF (30 ng/mL), RANKL (100 ng/mL), and the indicated concentration of MD35 for 7 days. The medium was changed every 72 h until the end of the 7-day culture period. The cells were removed by incubation with 5% sodium hypochlorite (Sigma-Aldrich, St. Louis, MO, USA) for 5 min. Resorption pits were observed under an optical microscope, and the percentage of resorption area in the total area was quantified using ImageJ software.

### Immunoblotting Analysis

To evaluate the effects of MD35 on the downstream signaling pathway for RANKL activation, BMM cells grown with M-CSF (30 ng/mL) were treated with RANKL (100 ng/mL) in the absence or presence of MD35 (50 μg/mL) for the indicated times. Cell lysates were resolved using 4–20% sodium dodecyl sulfate–polyacrylamide gel electrophoresis (Invitrogen, Paisley, UK) and transferred to nitrocellulose membranes (Amersham Bioscience, Westborough, MA, USA). Membranes were blocked with 3% skim milk before immunoblotting with a primary antibody (1:500 dilution) and horseradish peroxidase-conjugated secondary IgG (1:1000 final dilution). Signals were detected through enhanced chemiluminescence using Amersham ECL reagent and visualized with the ChemiDoc Imaging System (Azure Biosystems, Dublin, CA, USA).

### Animals

Female C57BL/6 mice were purchased from the Animal Facility of Aging Science of the Korea Basic Science Institute (KBSI) (Gwangju, Korea) and used in experiments at 6 weeks of age (25 − 30 g). Animals were housed in groups in a temperature-controlled room (20–22℃) under a 10-h dark, 14-h light cycle (lights on from 0600 to 2000 h) and provided food and water ad libitum. Two weeks after the operation, animals were randomly assigned to five groups: water control (sham, *n* = 6), OVX with water (OVX, *n* = 6), OVX with a low dose of MD35 (10 mg/kg, *n* = 6), and OVX with a high dose of MD35 (50 mg/kg, *n* = 6). Based on the concentration of postbiotic MD35 that suppresses osteoclast differentiation in vitro study, it was administered oral-fed in vivo at two different concentrations proportional to the animal's body weight. The mice received two different dosages, 10 mg/kg and 50 mg/kg of MD35, or water by oral administration once daily for 8 weeks. After 8 weeks, all mice were euthanized by CO_2_ inhalation and cervical dislocation. For histological and microarchitectural analyses, the femurs were fixed using 4% formaldehyde. All animals were maintained and treated in accordance with the National Institutes of Health Guidelines for the Care and Use of Experimental Animals; the animal experiments were approved by the Institutional Animal Care and Use Committee of the Korea Basic Science Institute, Gwangju, Korea (KBSI-IACUC-21–23).

### Micro-computed Tomography (Micro-CT) Analysis

After fixation, the mouse femurs were scanned to evaluate morphological characteristics using micro-computed tomography (Micro-CT) (Xradia Versa 620 imaging system, Zeiss, Dublin, CA), located at the Korea Basic Science Institute. The samples were scanned in custom-made sample holders that oriented the samples vertically on the stage and rotated horizontally by a 180° + fan. The femur was scanned at 10 μm resolution using a 0.4 × objective with energy settings of 60 kV, 108 μA, and a power source of 6.5 W with an air filter and 1201 projections. The structural images of the bone were presented using the Dragonfly Pro software (Object Research Systems, QC, Canada). The structural parameters for trabecular bone were analyzed using Analyze software (version 12.0; AnalyzeDirect, Overland Park, KS, USA). Bone mineral density of the femur was estimated using a hydroxyapatite (HA) phantom (QRM-MicroCT-HA, Quality Assurance in Radiology and Medicine GmbH, Germany) and scanned using the same parameters. Bone mineral density (BMD), bone volume (BV), bone volume fraction (BV/TV), trabecular thickness (Tb.Th), trabecular separation (Tb.Sp), and trabecular number (Tb.N) were calculated using ROI. For the growth plate measurement, the growth plate region was outlined and selected in the 2D and 3D growth plate slices, which were rendered into an area and volume for calculations. Growth plate thickness was measured using a ruler and histogram tool after cropping the growth plate volume into surfaces. The values of the parameters are presented as the mean ± Standard deviation (SD).

### Statistical Analysis

All quantitative data are presented as mean ± standard deviation. Statistical analysis was performed with GraphPad Prism 5 software (GraphPad Software, La Jolla, CA). The significance of differences was evaluated using a one-way analysis of variance (ANOVA) followed by Dunnett’s test for comparisons among multiple groups. A significant *p*-value in the figures is reported as the asterisks denote statistical significance (*, *P* < 0.05; **, *P* < 0.01; ***, *P* < 0.001).

## Results

### Postbiotic *L. plantarum* MD35 Inhibits Osteoclast Differentiation and Resorption Pit Formation In Vitro, but Does Not Affect the Viability of BMMs

**Fig. 1:**
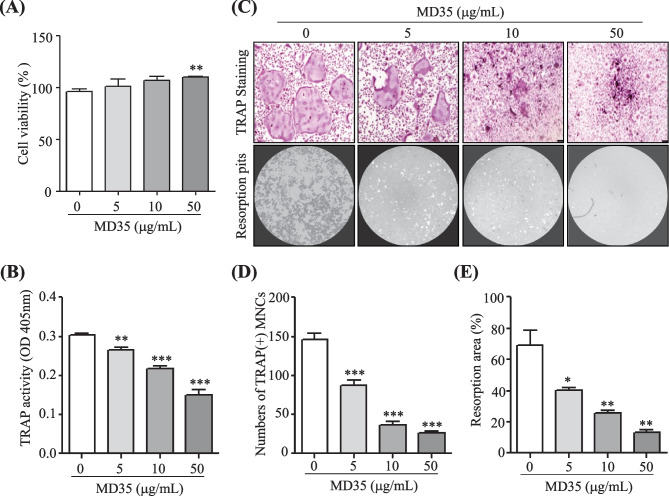
Effects of postbiotic *L. plantarum* MD35 on RANKL-induced osteoclast differentiation. **A** BMMs were cultured with M-CSF (30 ng/mL) in the indicated concentrations of postbiotic MD35 and cell viability was assessed for 3 days. Cell viability in the 0 μg/mL of MD35 was considered as 100%, and the percentage values for three different dosages (5, 10, and 50 μg/mL of MD35) were calculated. **B–E** BMMs were cultured in a tissue culture plate (**B**, **C** upper panel, and **D**) or in an Osteo Assay Surface plate (**C** lower panel and **E**) and treated with M-CSF (30 ng/mL) and RANKL (100 ng/mL) in the absence or the presence of an increasing concentration of postbiotc MD35 for 7 days. **B** The total cellular TRAP activity. (**C** Representative microscopic images of TRAP staining (upper panel) and resorption pits (lower panel). (**D** The number of TRAP-positive multinuclear cells (MNCs) with more than three nuclei. **E** Relative resorption area. Error bars represent the mean result ± SD of three independent experiments; *p < 0.05, ** *p* < 0.01, *** *p* < 0.001 vs. control

To investigate the effect of postbiotic MD35 from *L. plantarum* on the viability of BMMs, we performed cell viability assays for 3 days at increasing concentrations of MD35 (5, 10, and 50 μg/ml) and in the presence of M-CSF (30 ng/mL). Postbiotic MD35 had no effect on the viability of BMMs compared with that of the control (Fig. 1A). There were no effects on cell viability at concentrations up to 500 μg/ml (data not shown). Therefore, the postbiotic MD35 of *L. plantarum* was not cytotoxic to the BMMs. Next, we investigated the role of the postbiotic MD35 in osteoclast differentiation by analyzing TRAP activity in BMMs treated with M-CSF (30 ng/mL) and RANKL (100 ng/mL) in the absence or presence of an increasing concentration of the postbiotic MD35 for 7 days. TRAP activity is commonly used as an indicator of osteoclast differentiation. The postbiotic MD35 decreased the RANKL-induced TRAP activity in a dose-dependent manner (Fig. 1B). Furthermore, we investigated whether postbiotic MD35 suppresses RANKL-induced osteoclast formation and resorption activity. It significantly suppressed TRAP-positive osteoclast formation and the number of TRAP-positive multinuclear cells in a dose-dependent manner (Fig. 1C, upper panel, and D). In addition, the resorption pit area decreased with increasing concentrations of postbiotic MD35, compared to that of the control (Fig. 1C, lower panel, and D). Therefore, postbiotic MD35 inhibited osteoclast differentiation in BMMs, suggesting that it directly modulated osteoclast formation without inducing cytotoxicity.

### Postbiotic MD35 Inhibits the RANKL-Induced Signal Pathway

**Fig. 2:**
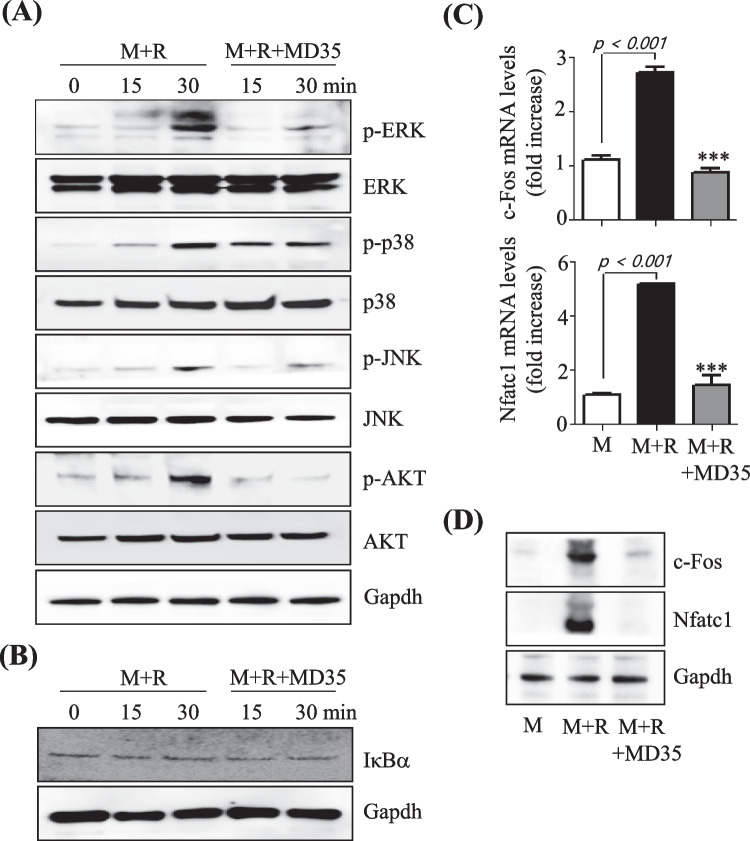
Effects of postbiotic MD35 on RANKL-induced signaling pathways and transcription factors. BMMs pre-treated with postbiotic MD35 (50 μg/mL) for 1 h were stimulated with M-CSF (M, 30 ng/mL) and RANKL (R, 100 ng/mL) for the indicated times. **A** Total lysates (30 ug/lane) were extracted and visualized using western blotting using p-ERK, p-p38, p-JNK, and p-AKT antibodies. **B** The expression levels of IκBα were determined using western blotting. Relative mRNA expression of Nfatc1 and c-Fos genes by qRT-PCR (**C**) and protein expression using western blotting analysis (**D**) using an antibody against Nfatc1 and c-Fos. Data are representative of three independently performed experiments. *** *p* < 0.001 vs. M + R. M, M-CSF; R, RANKL

To investigate the mechanism by which postbiotic MD35 suppresses RANKL-induced osteoclastogenesis, we examined its effect on the RANKL-induced early signaling pathways, including those involving nuclear factor-κB (NF-κB), three MAPKs including ERK, p38, and c-jun-N-terminal kinases (JNK), as well as activator protein-1 (AP-1) and Akt. Stimulation of BMMs with RANKL in the presence of M-CSF increased phosphorylation of p-ERK, p-p38, p-JNK, and p-AKT within 30 min. However, pre-treatment with postbiotic MD35 (50 μg/ml) for 1 h significantly reduced the phosphorylation of these signaling proteins (Fig. 2A). In contrast, postbiotic MD35 did not inhibit RANKL-induced degradation of IκBα protein (Fig. 2B), suggesting that there was no effect on NF-κB activating signaling. In BMMs, RANKL-inducing signaling pathways ultimately lead to the induction and activation of nuclear factor of activated T-cells cytoplasmic 1 (Nfatc1), a master transcription factor for osteoclast differentiation, via AP-1, c-Jun, and c-Fos. Therefore, to determine whether postbiotic MD35 inhibited RANKL-induced c-Fos and Nfatc1 expression, we measured mRNA and protein levels using qRT-PCR and Western blotting, respectively. Stimulation of BMMs with RANKL in the presence of M-CSF for three days increased the mRNA and protein levels of c-Fos and Nfatc1. However, pre-treatment with postbiotic MD35 (50 μg/ml) significantly reduced the mRNA and protein expression levels of c-Fos and Nfatc1 to control levels (Fig. 2C and D). Therefore, postbiotic MD35 suppresses MAPK/AP-1 signaling and c-Fos/Nfatc1 expression.

### Postbiotic MD35 Downregulates Osteoclastogenic Gene Expression

**Fig. 3:**
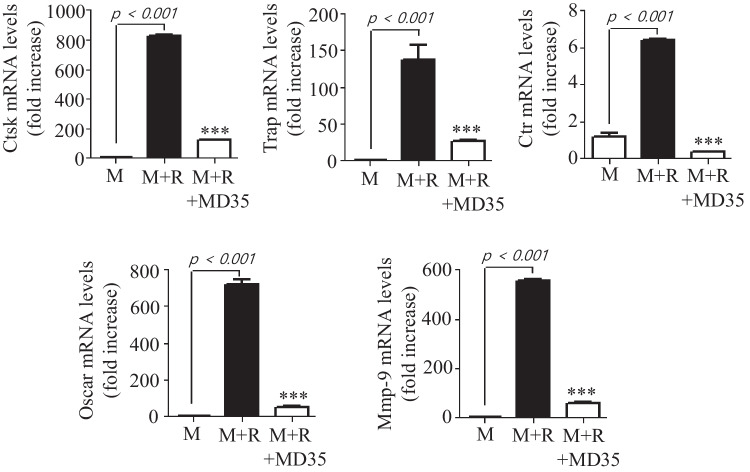
Effects of postbiotic MD35 on RANKL-induced osteoclastogenetic gene expression in BMMs. BMMs were cultured for 3 days with M-CSF (M, 30 ng/mL) and RANKL (R, 100 ng/mL) in the absence or the presence of an increasing concentration of postbiotc MD35. Total RNA was isolated from cell lysates, and qRT-PCR was performed using specific primers for the indicated genes. Each point on the graph are mean value from three independent experiments; *** *p* < 0.001 vs. M + R. M, M-CSF; R, RANKL

Postbiotic MD35 suppressed RANKL-induced osteoclast formation, by inhibiting Nfatc1 activation. Therefore, the effect on Nfatc1 target genes, such as Ctsk, Trap, Ctr, Oscar, and MMP-9, were investigated for their potential associates with osteoclast differentiation. Figure 3 shows that RANKL stimulation significantly induced the expression of these osteoclast-specific genes at the mRNA level, whereas postbiotic MD35 treatment inhibited the expression of these genes.

### Postbiotic MD35 Prevents Bone Loss in Ovariectomized Mice

**Fig. 4:**
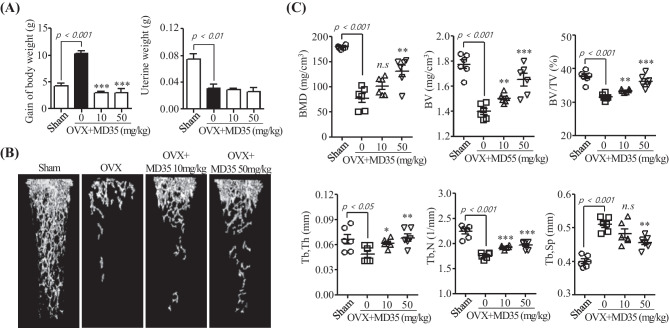
Effects of postbiotic MD35 on bone loss in mice osteoporotic models. **A** Alteration of body weight gain and uterine weight in sham or treatment without (0, water) or with two different dosages (10 and 50 mg/kg) of postbiotic MD35, through oral administration once daily for 8 weeks in ovariectomized (OVX) mice. **B** Representative 3D reconstruction using the micro-CT images of femurs from sham, OVX, postbiotic MD35-treated OVX mice group. **C** Bone parameters including bone mineral density (BMD), bone volume (BV), bone volume per total volume (BV/TV), trabecular thickness (Tb.Th), trabecular number (Tb.N), and trabecular separation (Tb.Sp) were analyzed. Data are presented as the mean ± SD. *n.s*, non-significant; * *p* < 0.05, ** *p* < 0.01, *** *p* < 0.001 vs. the OVX group. *n* = 6

To evaluate the in vivo effect of postbiotic MD35 on postmenopausal osteoporosis, we used an OVX mouse model. Eight weeks after OVX, the gain in body weight was significantly increased and uterine weight was decreased compared with that the sham surgery group. In contrast, oral administration of postbiotic MD35 (10 and 50 mg/kg) once daily for 8 weeks markedly suppressed OVX-induced body weight gain, however, it did not affect uterine atrophy (Fig. 4A). The 3D volume images of trabecular bone in OVX mice by a 3D viewer in micro-CT were dramatically reduced compared to those in sham surgery mice. However, it prevented the loss of trabecular bone following postbiotic MD35 administration in a dosage-dependent manner (Fig. 4B). Moreover, quantitative analysis of bone parameters revealed a significant reduction in BMD, BV, BV/TV, Tb.Th, and Tb.N, and an increase in Tb.Sp compared to those in the sham surgery mice (Fig. 4C). The trabecular bone microarchitecture of OVX mice was preserved by postbiotic MD35 administration in a dosage-dependent manner (Fig. 4C). Therefore, the postbiotic MD35 prevents postmenopausal osteoporosis by inhibiting osteoclast differentiation.

### Postbiotic MD35 Alleviated Destruction of Femoral Plate Growth in Ovariectomized Mice

**Fig. 5:**
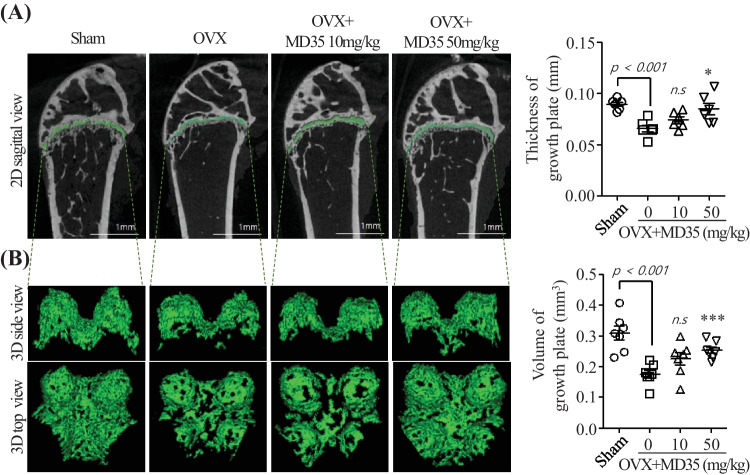
Effects of postbiotic MD35 on distal femoral growth plates in mice osteoporotic models. **A** In 2D images, the distal femoral growth plates are indicated in green; they were assessed by measuring at 5 points for the thickness in sham, OVX, postbiotic MD35-treated OVX mice group. **B** Growth plates of mice femur were generated from 3D reconstruction to calculate the growth plate volume in experimental groups; they are indicated in green. Data are presented as the mean ± SD*. n.s*, non-significant; * *p* < 0.05, *** *p* < 0.001 vs. the OVX group. *n* = 6

Postbiotic MD35 prevented the OVX-induced bone loss by inhibiting osteoclast differentiation. Therefore, we examined its effect on the epiphyseal growth plate in the femur, where bone formation occurs on a cartilaginous template. Using micro-CT analysis, the sagittal images and 3D volumetric rendering of the distal femoral growth plate were significantly lower in the OVX mice than in the sham surgery group (Fig. 5A and B, left panel). However, the structure (i.e., thickness, volume, and shape) of the femoral growth plate was restored by postbiotic MD35 administration in a dose-dependent manner. In addition, quantitative analysis revealed a significant decrease in growth plate thickness and volume in OVX mice compared to those in sham mice (Fig. 5A and B, right panel). The postbiotic MD35 markedly rescued these effects in a dose-dependent manner. Therefore, postbiotic MD35 prevents the destruction of femoral growth plates in postmenopausal osteoporosis.

## Discussion

Osteoporosis is a silent disease with no obvious symptoms until the occurrence of a bone fracture. Recently, the prevalence of osteoporosis has been increasing relative to the aging population, which is emerging as an important problem. Some supplements, such as calcium, vitamin D or K, as well as drugs such as selective estrogen receptor modulators, calcitonin, parathyroid hormone, other estrogen analogs, bisphosphonates, and hormone replacement therapy, are used to treat osteoporosis [9, 10]. However, long-term administration of these treatments causes several side effects such as osteonecrosis of the jawbone, fracture of the femur, and cancer [11, 22], therefore, new treatments with negligible side effects are needed.

Several prebiotics, probiotics, and postbiotics exhibit anti-osteoporotic activities [23–27, 30, 33–35]. In this study, we demonstrated the inhibitory effect of postbiotic *L. plantarum* MD35 on osteoclast differentiation in mouse bone marrow-derived macrophage (BMMs) culture in vitro. Moreover, postbiotic MD35 inhibited the RANKL-induced ERK, p38, JNK, and AKT phosphorylation, and downregulated the NFATc1 and c-Fos expression without affecting p65 expression during osteoclastogenesis. Furthermore, MD35 inhibited the RANKL-induced osteoclastogenic marker genes, Ctsk, Trap, Ctr, Oscar, and MMP-9, to regulate Nfatc1 gene expression. MD35 prevented bone loss in the femoral trabecular bone and growth plates in an OVX mouse model.

Research on the microbiomes in the human body has expanded to include multifaceted research on the diversity and roles of microorganisms in improving overall human health and preventing pathologies such as obesity, diabetes, inflammatory bowel disease, and osteoporosis. However, little is known regarding the role of therapeutic bacteria in bone health. Only a few studies have directly tested the effects of bacterial populations on the bone. These studies have focused on the acquisition of nutrients, such as calcium and phosphate, immune modulation, and direct effects through the production of small molecules, such as serotonin or estrogen-like molecules. Two different approaches have been explored: 1) treatment with a variety of microbiota, and 2) treatment with prebiotics or probiotics to directly deliver certain bacteria to the GI tract [23, 36]. The beneficial effects of prebiotics and probiotics on bone health have been previously demonstrated; however, the effects and mechanism of postbiotics from *L. plantarum* in menopausal osteoporosis have not been reported.

Osteoclasts differentiate from hematopoietic stem cells through induction of M-CSF and RANKL. The binding of RANKL to RANK on the surface of osteoclast precursor cells can stimulate diverse signaling pathways, including ERK, JNK, AKT, p38, and NF-κB, leading to the binding of several transcription factors, such as c-Fos, NF-κB, and Nfatc1 to the Nfatc1 promoter [13–16]. During osteoclast differentiation, Nfatc1 is a key regulator of several osteoclast-targeting genes such as Ctsk, Trap, Ctr, Oscar, and MMP-9 [19–21]. In this study, we demonstrated that the postbiotic, *L. plantarum* MD35, isolated from young radish water kimchi suppressed osteoclast differentiation, using TRAP activity assay, TRAP staining, and resorption pit formation assessment. Postbiotic MD35 suppressed RANKL-induced phosphorylation of ERK, p38, JNK, and AKT within 30 min; it had less effect on the RANKL-induced degradation of IκB. RANKL-stimulated mRNA and protein levels of Nfact1 and c-Fos are involved in Nfatc1 expression. However, it did not affect the mRNA or protein expression of p65 (data not shown). Therefore, postbiotic MD35 may downregulate RANKL-induced Nfatc1 expression by targeting MAPK/AP-1 signaling and Nfatc1 transcription via c-Fos/Nfatc1 expression. Further studies are needed to elucidate the precise molecular mechanism underlying the anti-osteoclast activity of the components of the postbiotic MD35 on Nfatc1 expression. A recent study reported that traditional Korean baechu kimchi could produce organic acids including lactic acid, fumaric acid, and acetic acid from kimchi cabbage waste [35, 37]. Among several acids, fumaric acid inhibits RANKL-mediated osteoclast differentiation [38], and these bioactive compounds may play a role in anti-osteoclastogenesis.

During osteoporosis development, indeed, the bone marrow mesenchymal stem cells represent differentiation into osteoblasts is inhibited and adipocytes are promoted. As a result, bone formation decreases, and the accumulation of bone marrow fat increases [39]. Due to increased bone marrow fat, bone healing, and regeneration are inhibited [40]. Indeed, an increased visceral fat mass is increased in women after menopause in human studies [41, 42]. As expected, the body weights of the six OVX mice were higher than that of the sham surgery group. Many fat tissues were present in the abdominal cavity of the OVX mice. In contrast, administration of postbiotic MD35 (10 and 50 mg/kg) orally, once daily, for 8 weeks markedly suppressed OVX-induced body weight gain, suggesting that postbiotic MD35 may have beneficial effects on reducing fat mass by inhibiting bone marrow fat accumulation. Furthermore, estrogen deprivation by OVX mice, whose ovaries were surgically removed, is often used to elucidate the effect of ovarian hormones on osteoporosis. In the present study, OVX-induced mice showed uterine hypotrophy and decreased uterine weight compared to those of the sham-operated group. However, it was no significant difference in uterine weight in OVX-induced mice with postbiotic MD35 treatment. Therefore, these data indicate that postbiotic MD35 did not affect hormonal regulation, especially estrogen production.

OVX animals exhibit estrogen deficiency, and are classically used as experimental models to simulate postmenopausal bone loss and increase osteoclastic activity after ovariectomy [24–27, 35]. Several studies showed that 3D Micro-CT images and histopathology assay represented significant bone loss after the OVX mice model. On the contrary, the estradiol-treated mice can restore OVX-induced bone loss and trabecular bone deterioration and showed increasing bone morphometric parameters, such as BMD, BV/TV, Tb.N, and Tb.Th [43–45]. We investigated the anti-osteoporotic effect of the postbiotic *L. plantarum* MD35 in osteoporosis in vivo by comparing osteoclastic activity and BMD in the OVX model. The body weights of the six OVX mice were higher than those of the sham-surgery group. Many fat tissue was present in the abdominal cavities of the OVX mice. In contrast, oral administration of postbiotic MD35 (10 and 50 mg/kg), once daily, for 8 weeks markedly suppressed OVX-induced body weight gain; however, it did not affect uterine atrophy. We investigated the cortical and trabecular femur to interpret the microstructural changes in the bone of OVX mice with and without postbiotic MD35 (0, 10, and 50 mg/kg). The in vivo model study results showed that postbiotic MD35 administration increased the volume and amount of trabecular bone as well as improved BMD, indicating that postbiotic MD35 has the potential to prevent bone loss in OVX mice.

Typically, growth plate measurements are performed to quantify growth plate heights using histomorphometry [46, 47]. Here, we introduced and applied quantitative 3D volume rendering from high-resolution micro-CT to establish accurate and reliable imaging for the detailed characterization of distal femoral growth plates in mice. The growth plate can be accurately depicted by reconstructing the micro-CT data (Fig. 5A and B). Postbiotic MD35 markedly rescued the growth plate volume and thickness in a dose-dependent manner. Therefore, we hypothesized that the postbiotic *L. plantarum* MD35 isolated from young radish water kimchi may attenuate bone loss by increasing BMD, BV, BV/TV, and Tb.N, reducing Tb.Sp, and alleviating the destruction of femoral plate growth in OVX mice.

Postbiotics have a longer shelf life, making them ideal substitutes for attractive compounds used in food production, and biological and pharmaceutical applications as well as long-term use as health promoters. Additionally, postbiotics are considered as safe as probiotics and have health benefits for humans as they exhibit several anti-inflammatory, immunomodulatory, anti-oxidant, and anti-osteoporotic properties. Therefore, postbiotic MD35 may have a therapeutic value in treating postmenopausal osteoporosis.

## Conclusions

In summary, the postbiotic *L. plantarum* MD35 exhibited a protective effect on osteoclast differentiation and bone resorption by modulating the MAPK/AP-1 signaling and Nfatc1 transcription via c-Fos/Nfatc1 expression after interacting with RANKL and RANK. Postbiotic MD35 further attenuated the bone loss in the femoral trabecular bone and growth plates in the OVX mouse model. Further studies are needed to demonstrate the mechanisms of components responsible for the antiosteoporotic effects shown by postbiotic MD35 and its effects on bone homeostasis.

## Data Availability

All data generated or analyzed during this study are included in this published article.

## Abbreviations

M-CSF: Macrophage colony-stimulating factor
RANKL: Receptor activator of the NF-κB ligand
BMM: Bone marrow-derived macrophage
Nfatc1: Nuclear factor-activated T cells c1
Ctsk: Cathepsin K
TRAP: Tartrate–resistant acid phosphatase
Ctr: Calcitonin receptor
Oscar: Osteoclast-associated receptor
MMP-9: Matrix metalloproteinase-9
